# Study of particle number size distributions at Azadi terminal in Tehran, comparing high-traffic and no traffic area

**DOI:** 10.1016/j.mex.2018.11.013

**Published:** 2018-11-27

**Authors:** Ramin Nabizadeh, Mahmood Yousefi, Faramarz Azimi

**Affiliations:** a Department of Environmental Health Engineering, School of Public Health, Tehran University of Medical Sciences, Tehran, Iran; b Center for Air Pollution Research (CAPR), Institute for Environmental Research (IER), Tehran University of Medical Sciences, Tehran, Iran

**Keywords:** PM, Traffic, Size distribution, Iran

## Abstract

Vehicle traffic is known as the anthropogenic aerosol source in megacities. Exposure to ambient air pollution, especially particulate matter has become the most environmental risk factor. The main aim of this study is to determine the particle number and their size distribution in Tehran at Azadi terminal (located in the West of Tehran), crossing of Nawab and Azadi streets the area with high traffic, and campus of Tehran University as an area without traffic. Particle size distribution (0.3–1 μm) was measured using a Grimm Environmental Dust Monitor and was conducted in two seasons, hot and cold (summer 2016 and winter 2016). The measurement was performed twice per month. Although the average number of particles at Azadi Terminal was more than the other two locations in both seasons but it was not significant) p > 0.05). The average number of particles larger than 0.3 μm was 286.72 ± 129.55 and 183.61 ± 86.79 cm^−3^ in winter and summer respectively. In relation to particles size distribution, the average number of particles larger than 0.4, 0.5, 0.65, 0.8 and 1 μm in winter and summer were 111.5 ± 120, 29.3 ± 23.7, 8.2 ± 5.8, 4 ± 3, 2 ± 1.5 and 52.5 ± 37, 14.4 ± 10.8, 6.1 ± 5, 3.8 ± 3.5, 2.3 ± 2 cm^−3^ respectively. In the current study the highest number of particles significantly observed in winter time in comparison to summer. In addition, had no significant difference between the number of particles at three sampling locations.

## Graphical abstract

**Figure:**
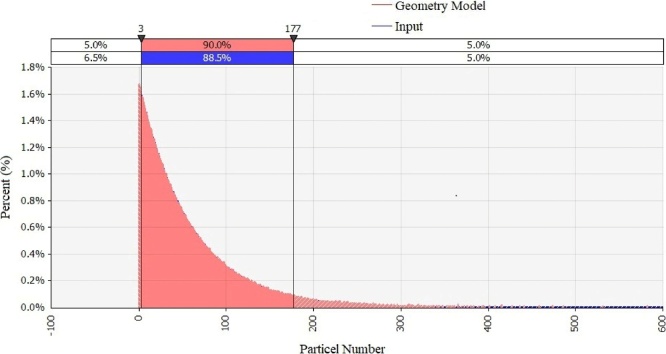

## Introduction

Particulate matters are significant air pollutants which release from a mixture of natural and man-made sources. A variety of heavy metals were found in particulate matter’s structure. These heavy metals which are mainly emitted from vehicles, are one of the main air pollutants having great economic and health impact. Nanoparticles cause health hazards especially in areas where geographical condition reduces natural ventilation [[1], [2], [3]]. Tehran is a high-altitude city by a population of near 8 million people and has an area of about 2300 square kilometers. The population is greatly at risk from air pollution, especially particulate matters. The city is surrounded by mountains with an altitude of about 3800–1000 m in North-South and East which exacerbates pollution in the city [[4], [5], [6], [7]]. Additional factors such as fast urbanization, uncontrolled emissions from cars and lack of infrastructures have caused a drop in the air quality in Tehran [8]. Vehicles considered the main cause of air pollution in Tehran. About 2 million automobiles with over 20 years of age are commuting daily and cause large emissions of particulate matter [9]. According to Tehran Air Quality Control Company reports since the beginning of 2016 to the end of this year, there were only17 days with clean air, 261 days of healthy air, 80 days were unhealthy for sensitive groups and 9 days were unhealthy for everyone. The reports show that other pollutants have little effect on the air quality of Tehran, for instance, nitrogen compounds were responsible for pollution for only 3 days throughout the year and ozone caused pollution only for one day during the said time. Particulate matters (mostly particles of less than 2.5 μm) are the cause of unhealthy air for the remaining days. Several studies have shown that there is a relation between health effects of particulate matters and their characteristics such as size, distribution, mass concentration and number concentration. This information can even be considered the best predictor of health consequences associated with the particles [10,11]. Most of the particles are categorized in the ultrafine group which contains particles that are less than 0.1 μm in diameter, known as ultrafine particles. Ultrafine particles have the largest surface area and the lowest mass, thus they have minimal impact in standard method of measuring particles by volume weight while having significant health effects and can easily enter the bloodstream through respiratory system walls and create many problems [12,13]. Due to slow sedimentation rate of particles with a size range between 0.1–1 μm, they can remain in the air for a long time and therefore have an increased risk of entering the body [14,15]. Size distribution of atmospheric particles with their ingredients and identifying their sources are known as key elements in management of health effects and climate change. Effects of smaller particles on health, is an interesting subject for many researchers. A great deal of research has been carried out on this subject and so fat the results have shown that in terms of mass concentration smaller particles have more health effects [16,17]. To the interest in measuring particles size distribution has led to measurement of the distribution of the particles in many cities and research centers [[18], [19], [20], [21]]. A survey conducted by Aron and Jain in 2007 in Delhi, India cleared that the main contributing particles were those with diameters less than 0.7 μm. Higher concentrations of heavy metals also happens in this range [22]. In a study by Samara and Voutsa in 2005 in northern Greece, the concentrations of heavy metals were detected in the following range: <0.8, 0.8–1.3, 1.3–2.7, 2.7–6.7 and >6.7 μm. Approximately this study had the same results with the results of the study conducted in Delhi and the highest concentration of particles was seen in the range of less than 0.8 μm. The major pollution sources were defined as traffic jam, industrial activities and resuspension of sediments [14]. Watson et al in a study at the University of Birmingham focused on particles number and size distribution in 2015 and studied sources of particulate emissions including traffic, industrial, biomass burning, cooking, traveling and marine aerosols. Based on the findings, size and number distribution of particles in an urban area is mostly affected by traffic emissions [23]. Although numerous studies in Tehran have focused on the particle mass concentration, their chemical characterizations and the association between particulate matter and their health effects [[24], [25], [26], [27]]. Studies that show the size and number distribution of each particle fractions are relatively scarce. In reality, size and number distribution of each particle fractions in this megacity are still unclear. Therefore, the principal objective of this study was to investigate the number concentration in three locations, including Azadi terminal, crossing of Nawab and Azadi street, and campus of Tehran University.

## Materials and methods

### Sampling location and study area

This study was conducted in Tehran as the populous city and capital of Iran. Tehran has specific geographic conditions, with the Alborz Mountains in the north. Additionally, the annual mean temperature based on daily data is 18.5 ℃. In addition, the highest and lowest temperature is found in July (43 ℃) and January (-15℃) respectively. The annual average of precipitation is 220 mm, with the maximum in winter time, March (approximately 40 mm), and the minimum in summer time, September (1 mm). Typically, the weather is sunny in Tehran. The annual average of bright sunshine and cloud cover is 2800 h and 30% [28]. One of the areas where particles measurement took place, was Azadi terminal, located on the northwest side of Azadi Square and at the beginning of Karaj Special Highway (Shahid lashgari Highway) with an area of 50 ha. Buses take passengers to cities in western and southwestern of Iran from this terminal. There are also buses for the main squares of Tehran and near cities and townships. The next location is crossing of Nawab and Azadi street where there's a lot of traffic during both the day and the night and is considered as a high-traffic area. Another sampling location was the Tehran University as an area with low traffic.

### Sampling and instrumentation

Particle size distribution in the range of 0.3 to 1 μm was measured using a Grimm Environmental Dust Monitor (EDM, model 107, Grimm GmbH, Ainring, Germany) and due to the impact of temperature on number of particles exhausted from cars, this study was conducted in two seasons, hot and cold (Summer 2016 and winter 2016) and the measurement was performed twice per month. The sampling started at 9:00 a.m. and ended at 14:00 p.m. Additionally, the measurements were separately at three sampling locations. It should be noted that duration for each measurement was only 60 min. According to the time interval set by the device, the number of particles per liter of air was measured every five minutes. In total 6 measurements for the warm and 6 times for cold season (every two weeks) were performed. At each location, the dust monitor was located at 1.5 m above the ground.

### Data analysis methods

In this study, we used generalized linear model by R software version 3.3.2 to analyze particles counts in relation to particles size class, season and sampling location.

## Results

### Particles number and size distribution

Results include data from summer 2016 and winter 2016. Table 1 gives the statistics of number concentrations in different size ranges from 0.3 to 1 μm. The following six sub-size ranges were divided into< 0.3, < 0.4, < 0.5 > 0.65, < 0.8 and < 1 μm.

**Table 1:** Descriptive statistics of particle number in different size bins.

Location	Size	Mean (cm^−3^)	Median (cm^−3^)	Max (cm^−3^)	Min (cm^−3^)	SD (cm^−3^)
Summer						
Azadi terminal	0.3	231	154	517	148	91
	0.4	66	43	141	41	42
	0.5	20	14	45	13	13
	0.65	9	6	21	5	6
	0.8	6	4	14	2	4
	1	3	2	8	1	3
University of Tehran	0.3	125	78	363	66	117
	0.4	35	22	99	18	31
	0.5	9	6	25	4	8
	0.65	3	2	10	2	3
	0.8	2	1	6	1	2
	1	1	1	4	0	1
Four Ways Navab	0.3	195	139	450	116	130
	0.4	56	40	128	33	37
	0.5	15	11	35	9	10
	0.65	6	4	16	4	5
	0.8	4	3	11	2	3
	1	3	2	7	1	2

Winter						
Azadi terminal	0.3	317	308	563	67	168
	0.4	105	103	201	18	60
	0.5	29	29	57	6	18
	0.65	9	7	17	3	5
	0.8	5	3	11	2	4
	1	2	2	7	1	2
University of Tehran	0.3	263	218	573	47	187
	0.4	138	76	402	13	147
	0.5	34	18	97	3	36
	0.65	9	7	22	1	8
	0.8	4	3	9	1	3
	1	2	1	4	1	1
Four Ways Navab	0.3	280	297	478	47	151
	0.4	91	98	176	13	55
	0.5	24	25	49	4	15
	0.65	7	6	13	2	5
	0.8	4	3	9	1	3
	1	2	2	4	1	1

A summary of the number of particles in each sampling location at seasons is shown in Table 1. The average number of particles per cm^3^ in air in Azadi Terminal was more than the other two situations in both season and the University of Tehran had the smallest amount of particles per cm^3^ of the air. Generalized linear model was used to compare models which will be shown below.

### Normality of the distribution particle number

**Fig. 1:**
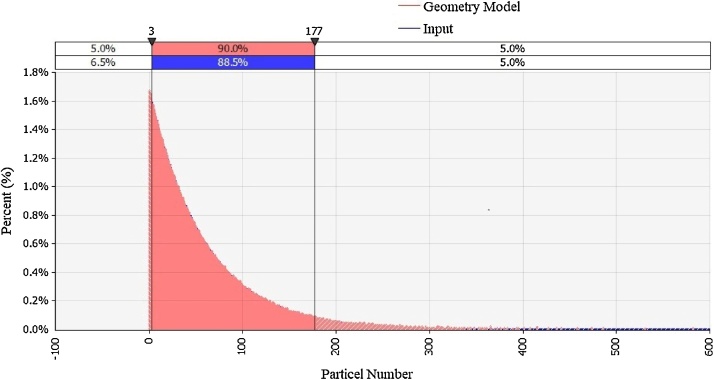
Particle number per cm^3^.

Normality of the distribution of the particle numbers was checked through Shapiro-Wilk test of normality. The results showed which particles do not have normal distribution at the 99% conﬁdence level (p < 0.01) and therefore it was necessary to transform the original data through their natural logarithms. In the next step, we showed the distribution particle number range of 0.3 up to 1 μm using the Risk Analysis Software (Fig. 1).

Fig. 1 shows the geometry of particle number in our study. Our findings revealed that

As shown in Fig. 1, 90% of the data refer to the number of the 3–177 per cm^3^ and the data has skewness to left and does not show normal distribution.

Generalized linear model is used to model data that do not have a normal distribution. The most important feature of this model is that it relates one or more dependent continuous variables (particle number cm^−3^) to one or more independent variables (season, place of measurement and particles classification). Finally, to compare the variables influencing the number of particles released to the atmosphere was used the generalized linear model and variance analysis.

According to Table 2, the season and particles size distribution affected the number of particles was measured (P < 0.001) and location not significant (P > 0.05). Therefore, the investigated variables are significant and should be compared meaningfully. At this stage, we used the ANOVA test. After that, Tukey was used as the post hok test. According to the results of Tukey test, there is a significant difference between all particle size fractions (P < 0.005). The average number of particles larger than 0.3 μm was 286.72 ± 129.55 and 183.61 ± 86.79 cm^−3^ in winter and summer respectively. In relation to particles size distribution, the average number of particles larger than 0.4, 0.5, 0.65, 0.8 and 1 μm in winter and summer were 111.5 ± 120, 29.3 ± 23.7, 8.2 ± 5.8, 4 ± 3, 2 ± 1.5 and 52.5 ± 37, 14.4 ± 10.8, 6.1 ± 5, 3.8 ± 3.5, 2.3 ± 2 cm^−3^ respectively. A study in Helsinki showed that the number of particles was inversely related to air temperature. Also, the number of smaller fraction of particles in wintertime was significantly higher than that of in summer owing to occurring inversion phenomenon in late autumn and early winter and incomplete combustion. The result of our study is in line with Hussein [29]. Based on the results of GLM for sampling locations, there was no significant difference between three locations (P > 0.05). The main reason for this is the fact that Tehran has a specific spatial traffic with more than 4 million vehicles that mainly are outdated. In fact, the air pollutant-related to traffic affect all districts of Tehran [30].

**Table 2:** The results of the Generalized linear model for particle number by Season, Location and size distribution.

Variable	Df	Deviance	AIC	F value	Pr(>F)
Season	1	6448.9	7416	30.8910	8.226e-08 ***
Location	2	5755.7	6720.9	2.5001	0.08452.
size distribution	1	26623.6	27590	784.4978	2.2e-16 ***

Sig. Codes : 0 ‘***’ 0.001 ‘**’ 0.01 ‘*’ 0.05 ‘.’ 0.1 ‘’ 1.

**Fig. 2:**
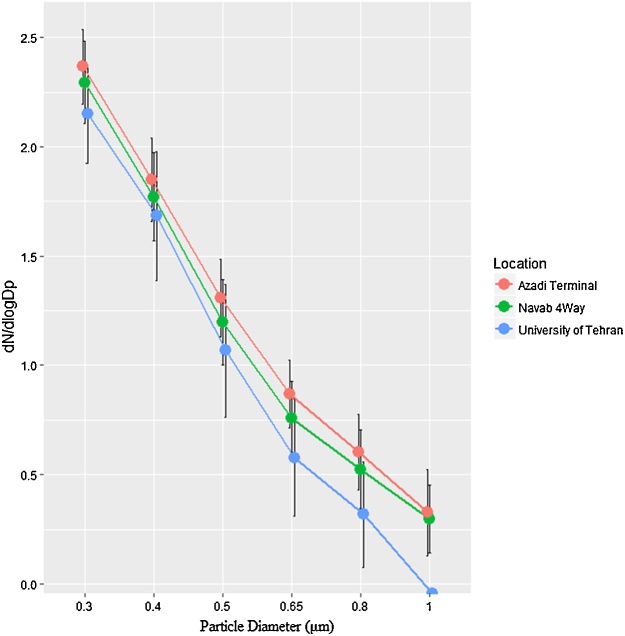
Mean plot of log normality distribution particles in three sampling locations.

Fig. 2 compares mean plot of lognormality distribution particles in three sampling locations. As shown in Fig. 2, the highest number of particles for all classes of particles was found at Azadi terminal. Furthermore, the results of our study revealed that the number of particles significantly decreased with increasing the particle diameter. Exposure to ambient particle size fractions has become a leading environmental risk factor in global megacities in particular in developing countries. A study in Tehran revealed that short-term exposure to particle size fractions has a positive association with inflammatory biomarkers such as white blood cells, high sensitive C-reactive protein (hsCRP), tumor necrosis factor-soluble receptor-II (sTNF-RII), interleukin-6 (IL-6), and von Willebrand factor in elderly subjects [31]. Amongst mentioned biomarkers, CRP, IL-6 and sTNF-RII act as predictors of cardiovascular mortality and morbidity [32]. Furthermore, a study in the Brisbane metropolitan area, Australia, showed that the number of particles was positively associated with an increase in hsCRP and exhaled nitric oxide (FeNO) among children aged 8–11 years [33].

## Conclusion

In summary, we examined the particle size fractions at three locations in Tehran during two seasons. According to the results of the current study, the highest number of particles measured was in the range of 0.3–0.4 μm. In this study, according to the model, there were significant differences between seasonal results. In relation to the number of particles in two seasons, the number of particles larger than 0.3 μm in winter was statistically signiﬁcantly higher than that in summer due to the effect of incomplete combustion of vehicles and the inversion phenomenon in winter. It should be noted that ambient particles, as the most important air pollutants, are an inseparable part of the human societies. To protect public health, particularly susceptible groups, appropriate sustainable control strategies and policies are recommended.

## Conflict of interest

The authors of this article declare that they have no conflict of interests.
